# PReS-FINAL-2167: What is the potential of intra-articular corticosteroid injections to induce sustained remission in children with juvenile idiopathic arthritis?

**DOI:** 10.1186/1546-0096-11-S2-P179

**Published:** 2013-12-05

**Authors:** S Lanni, F Bovis, C Papadopoulou, M Kostik, MI Gonzalez-Fernandez, M Bohm, JC Nieto-Gonzalez, M Bertamino, A Martini, A Ravelli

**Affiliations:** 1 IRCCS Istituto Giannina Gaslini, Genova, Italy; 2 University of Genova, Genova, Italy

## Introduction

Intra-articular corticosteroid injections (iacis) are widely used in the management of children with juvenile idiopathic arthritis (JIA). Although this therapeutic intervention is generally considered for the treatment of children with arthritis in a small number of joints, the strategy of injecting simultaneously multiple joints has been advocated in children with polyarthritis. However, the relative indications of single and multiple iacis in the management of JIA remains unclear and controversial. Furthermore, the capacity of iacis to induce sustained remission of synovitis in injected joints is still poorly documented.

## Objectives

To describe our 10-year experience on the use of single and multiple iacis in the management of children with JIA.

## Methods

The clinical charts of 542 JIA patients who received an IACI in 1,2 or ≥ 3 joints between January 2002 and December 2011 and had a follow-up duration of at least 6 months after the procedure were reviewed. The corticosteroid preparation used was triamcinolone hexacetonide for large joints and methylprednisolone acetate for small or difficult to access joints. For each patient, the follow-up period after the IACI was censored when one of the following events occurred: 1) flare of synovitis in injected joints; 2) flare of synovitis in injected and uninjected joints; 3) follow-up visit with continued remission of synovitis in injected joints, but recurrence of synovitis in uninjected joints; 4) last follow-up visit with continued remission of synovitis in both injected and uninjected joints. For the purposes of the analysis, events 1) and 2) were considered together and defined as "flare of synovitis in injected joints", whereas events 3) and 4) were considered together and defined as "remission of synovitis in injected joints".

## Results

Two hundred and fifteen (39.7%) patients were injected in 1 joint, 107 (19.7%) were injected in 2 joints and 220 (40.6%) were injected in ≥ 3 joints. Following IACI therapy, 138 (25,5%) patients were in remission at last follow-up visit, whereas 120 (22.1%), 93 (17.2%), and 191 (35.2%) experienced a flare of synovitis, respectively, in uninjected joints, injected joints and either injected and uninjected joints. The cumulative probability of survival without synovitis flare for patients injected in 1, 2, or ≥ 3 joints was 69, 46, 49%, respectively, at 1 year; 59, 31, 30%, respectively, at 2 years; and 40, 21, 19%, respectively, at 3 years.

## Conclusion

IACI therapy, either in single or multiple joints, was able to induce sustained remission of synovitis in a sizable number of patients. Our findings indicate that iacis remain a mainstay in the management of children with JIA, even in the current biologic era.

## Disclosure of interest

None declared.

